# The prospective relationship between sedentary time and cardiometabolic health in adults at increased cardiometabolic risk – the Hoorn Prevention Study

**DOI:** 10.1186/s12966-014-0090-3

**Published:** 2014-07-16

**Authors:** Teatske M Altenburg, Jeroen Lakerveld, Sandra D Bot, Giel Nijpels, Mai JM Chinapaw

**Affiliations:** 1 Department of Public and Occupational Health, VU University Medical Center, EMGO Institute for Health and Care Research, Amsterdam, The Netherlands; 2 Department of General Practice and Elderly Care Medicine, VU University Medical Center, EMGO Institute for Health and Care Research, Amsterdam, The Netherlands

**Keywords:** Screen time, Weight indicators, Cardiovascular risk factors, Fasting blood samples

## Abstract

**Background:**

Sedentary time has been identified as an important and independent risk factor for the development of type 2 diabetes mellitus (T2DM) and cardiovascular diseases (CVD) in adults. However, to date most studies have focused on TV time, few also included other sedentary behaviours such as computer use and reading, and most studies had a cross-sectional design. We aimed to examine the prospective relationship between time spent on sedentary behaviours in different domains with individual and clustered cardiometabolic risk in adults.

**Methods:**

Longitudinal data of 622 adults aged 30-50 years (42% males) at increased cardiometabolic risk were used. Leisure time TV viewing, computer use, reading and other sedentary activities (e.g. passive transport) were assessed using a subscale of the Activity Questionnaire for Adolescents and Adults (AQuAA), and summed into overall sedentary behaviour (min/day). Weight and blood pressure were measured, waist-to-hip ratio and BMI calculated, and fasting plasma levels of glucose, HbA1c, total cholesterol, HDL-cholesterol, LDL-cholesterol and triglycerides determined. T2DM risk score was estimated according to the ARIC formula and CVD mortality risk according to the SCORE formula.

**Results:**

Generalized Estimating Equation analysis demonstrated that over a two-year period higher levels of overall sedentary time and TV time were weakly but negatively associated with one out of 13 studied cardiometabolic risk factors (i.e. HDL cholesterol).

**Conclusion:**

Overall sedentary time, as well as sedentary time in different domains, was virtually not related with cardiometabolic risk factors.

## Background

Sedentary behaviour refers to activities performed sitting that typically have low energy expenditure (1 to 1.5 metabolic equivalent multiples of rest) [1]. Recently, sedentary behaviour has been identified as an important and independent lifestyle risk factor of type 2 diabetes mellitus (T2DM) and cardiovascular disease (CVD) [2]–[6].

The hypothesised mechanism underlying the biological consequences of prolonged sitting suggests that loss of local contractile stimulation in weight bearing muscles leads to the suppression of skeletal muscle lipoprotein lipase (LPL) activity [7],[8]. The loss of LPL activity at the vascular endothelium impairs several aspects of lipid metabolism [9], and may contribute to increased cardiometabolic risk.

A number of reviews demonstrated mixed findings for a positive longitudinal relationship of overall sedentary time with the risk of T2DM and CVD [5],[6]. Most studies have focused on TV time only and the few studies that examined the association of sedentary behaviours other than TV time with individual and clustered cardiometabolic risk, reported mixed findings [10]–[14]. All these studies found a significant positive association of TV time with individual (i.e. waist circumference, body mass index, plasma levels of triglycerides, cholesterol, insulin) and clustered (i.e. obesity risk, T2DM risk) risk [10]–[14]. Contradictory results were found for the associations of other sedentary behaviours such as computer use, sitting at work, reading and passive transport with cardiometabolic risk [10]–[14].

There is a need for methodological sound prospective studies on sedentary activities – especially including other activities than TV viewing – and cardiometabolic risk factors. In order to develop effective interventions preventing chronic diseases, it is important to examine whether specific sedentary activities should be targeted. Therefore, the present study examined the prospective associations between self-reported sedentary time in different domains (i.e. TV viewing, computer use, reading and other sedentary activities) and cardiometabolic risk factors, including four follow up measurements over a period of 2 years in adults (aged 30-50 years) at increased cardiometabolic risk.

## Methods

### Study design and participants

The adults selected for this study were participants of the Hoorn Prevention Study, which was carried out in the Netherlands from December 2007 to May 2010, with the purpose of evaluating a cognitive behaviour program aimed at preventing T2DM and CVD [15]. Briefly, a total of 8193 men and women aged 30-50 years and living in the semi-rural region of West Friesland, the Netherlands, were invited to self-report their waist circumference, following detailed written instructions. Of the 3587 respondents (43.8%), 2401 indicated that they were willing to participate. Of the 921 participants with an unhealthy waist circumference, 722 visited the Diabetes Research Center for baseline measurements, gave written informed consent and participated in the trial. A total of 622 men and women, aged 30-50 years, having at least a 10% estimated T2DM risk and/or 10% estimated CVD mortality risk and no known prevalent T2DM or CVD were randomly assigned to either the intervention group or the control group of the Hoorn Prevention Study. Of the 622 participants, 79% of participants attended the last follow up measurement at 24 months. Drop-out analysis showed no differences in baseline values of ARIC and SCORE risk formulas between participants who completed the study and those who dropped out (*T*-test ARIC p = 0.10 (95% CI −3.63 – 0.33); SCORE p = 0.99 (95% CI −0.60 – 0.59)).

The cognitive behaviour programme was not effective in improving cardiometabolic risk, lifestyle behaviours or psychological determinants of lifestyle behavioural change on the short, medium or long term [16]–[18]. Therefore, data from both the intervention and the control group were included in the present analysis.

### Measurements

#### Cardiometabolic risk

Biomarkers of cardiometabolic risk included indicators of overweight (i.e. body weight, waist- and waist-to-hip ratio), blood pressure, plasma levels of fasting glucose, insulin glycated haemoglobin (HbA1c), total cholesterol, high-density lipoprotein cholesterol (HDL-C), low-density lipoprotein cholesterol (LDL-C) and triglycerides. The 9-year risk of developing T2DM and the 10-year risk of a fatal CVD were estimated according to the formula described in the Atherosclerosis Risk In Communities (ARIC) study [19] and the Systematic COronary Risk Evaluation (SCORE) project [20].

Weight was measured to the nearest 0.5 kg, wearing light clothes and no shoes. The standard scales that were used (SECA; London, UK) were calibrated yearly. Waist circumference was measured midway between the lowest rib margin and the iliac crest, and hip circumference at the level of the iliac crest. Two measurements to the nearest 0.5 cm were recorded for both waist and hip; if the difference between the measurements was greater than 1 cm, a third measurement was carried out and the mean of the two nearest measurements was calculated. Systolic and diastolic blood pressure were measured three times on the right arm after 10 minutes of rest, in a seated position, with a Colin Press BP 8800p Non-Invasive Blood Pressure Monitor (Colin Medical Technology Corporation, USA). Mean systolic and diastolic blood pressure were calculated as the mean of the last two measurements. Fasting plasma glucose was measured according to the enzymatic reference method with hexokinase, HbA1c determination was based on the turbidimetric inhibition immunoassay for haemolysed whole blood, and total and HDL-C and triglycerides were measured with the enzymatic colorimetric method. All laboratory tests were performed using the Cobas Integra system (Roche diagnostics, Basel, Switzerland). The ARIC formula was based on ethnicity, parental history of diabetes, systolic blood pressure, waist circumference, and height. The SCORE formula included sex, smoking status, total cholesterol, and systolic blood pressure.

#### Sedentary time

Sedentary time during leisure in minutes per day (weekdays and weekend days combined) was assessed using a subscale of the Activity Questionnaire for Adolescents & Adults (AQuAA), which has been tested for reliability and validity [21]. In separate questions, participants were asked how many days in the last week and how many time per day they spent on average 1) watching TV, 2) using the computer, 3) reading, and 4) on other sedentary activities (e.g. sitting while talking with friends, playing board games, sitting in the car). Total sedentary time was calculated by summing the minutes per day spent in the different domains of sedentary behaviour.

#### Covariates

The following socio-demographic and lifestyle covariates were considered as confounders: age, gender, education level (low, medium or high), parental T2DM (at least one parent with diagnosed T2DM or not), cigarette smoking (smoking regularly or not), total physical activity and intervention group (yes/no). Total physical activity was assessed using the validated Short QUestionnaire to ASsess Health-enhancing physical activity (SQUASH) [22]. Participants were asked to report time spent in light, moderate and vigorous physical activity during commuting, leisure-time, sports, household and work during one (regular) week in the past month. Total physical activity (light, moderate and vigorous) was calculated by summing the minutes per day spent in the different domains.

### Statistics

Descriptive participant characteristics (mean (SD)) and median (interquartile range) were calculated at each time point (i.e. at baseline and after 6, 12 and 24 months). Generalized Estimating Equations (GEE) with an exchangeable correlation structure were used to assess the prospective association between self-reported sedentary time and cardiometabolic risk factors (Model 1). This longitudinal analysis technique was used to adjust for dependency within the repeated measures for each participant, by capturing the changing status of sedentary time and cardiometabolic risk, as well as the relationship between them, over time [23]. Age, gender, education level and total physical activity considerably confounded the associations of sedentary time with cardiometabolic risk factors and were therefore included in the analysis (Model 2).

In addition to the association of overall self-reported sedentary time, the prospective associations of TV time, computer time, reading time and time spent on other sedentary behaviours with cardiometabolic risk factors were assessed separately. In the latter analyses, next to adjustments for age, gender, education level and total physical activity, these associations were adjusted for time spent sedentary in the other domains. All statistic procedures were performed using SPSS software (version 20.0). The significance level was set at P < 0.05.

## Results

Table 1 shows participant’s cardiometabolic risk factors, their sedentary time and time spent on physical activity. At baseline, participants (42% males) were on average 43.5 (5.3) years old and 21% were regular smokers. Education level was low for 33%, medium for 46% and high for 21% of the participants. Median values for time spent on all recalled sedentary activities combined were 255, 253, 229 and 232 min/day at baseline and after 6, 12 and 24 months, respectively.

**Table 1:** Participant characteristics

	**Baseline**	**After 6 months**	**After 12 months**	**After 24 months**
	**N = 609-622**^**a**^	**N = 524-536**^**a**^	**N = 500-504**^**a**^	**N = 479-491**^**a**^
**Cardiometabolic risk factors (mean (SD))**				
Weight, kg	90.5 (15.5)	90.3 (15.1)	90.4 (14.9)	90.1 (15.1)
WC, cm	96.7 (9.8)	96.3 (9.6)	96.0 (10.5)	96.0 (9.9)
Waist-to-hip ratio	0.90 (0.08)	0.90 (0.08)	0.90 (0.08)	0.90 (0.08)
SBP, mmHg	129.0 (13.2)	128.0 (13.3)	127.1 (15.0)	126.7 (13.1)
DBP, mmHg	73.4 (9.5)	72.6 (9.5)	71.5 (9.2)	72.2 (9.7)
Glucose, mmol/l	5.3 (0.5)	5.5 (0.5)	5.5 (0.5)	5.5 (0.5)
HbA1c, mmol/l	5.5 (0.3)	5.5 (0.3)	5.5 (0.3)	5.6 (0.3)
Cholesterol, mmol/l	5.5 (1.0)	5.5 (0.9)	5.5 (0.9)	5.5 (1.0)
HDL-C, mmol/l	1.3 (0.3)	1.3 (0.3)	1.3 (0.4)	1.4 (0.4)
LDL-C, mmol/l	3.6 (0.9)	3.5 (0.9)	3.6 (0.9)	3.5 (0.9)
Triglycerides, mmol/l	1.5 (0.9)	1.6 (0.8)	1.6 (0.9)	1.5 (0.9)
ARIC	18.9 (8.2)	18.4 (8.1)	18.1 (8.8)	18.0 (8.0)
SCORE	3.9 (3.0)	3.9 (3.0)	3.8 (3.9)	3.7 (3.0)
**Sedentary time and time spent on physical activity (median (interquartile range; 25-75))**				
Sedentary time, min/day	255 (171-315)	253 (154-304)	229 (150-284)	232 (150-287)
TV viewing, min/day	120 (60-150)	93 (60-146)	90 (51-127)	90 (60-129)
Computer use, min/day	26 (9-60)	30 (11-60)	26 (11-60)	30 (13-60)
Reading, min/day	21 (9-40)	21 (9-34)	23 (9-40)	21 (9-39)
Other SB, min/day	43 (21-85)	43 (17-85)	39 (17-69)	34 (17-67)
Total PA, min/day	386 (277-467)	396 (268-482)	381 (275-470)	379 (266-459)

^a^Due to missing data, sample sizes varied at the different time points.

ARIC, Atherosclerosis Risk In Communities; DBP, diastolic blood pressure; HbA1c, glycated haemoglobin; HDL-C, high-density lipoprotein cholesterol; LDL-C, low-density lipoprotein cholesterol; PA, physical activity; SB, sedentary behaviour; SBP, systolic blood pressure; SCORE, Systematic COronary Risk Evaluation; WC, waist circumference.

Table 2 shows the prospective association of overall self-reported sedentary time with cardiometabolic risk factors, for the crude (Model 1) and the adjusted (Model 2) associations.

**Table 2:** Prospective association (Beta and 95% CI) between self-reported overall sedentary time (h/day) and cardiometabolic risk factors

	**Model 1**	**Model 2**
	**Beta [95% CI]**	**Beta [95% CI]**
**Weight, kg**	0.30 [-0.05; 0.11]	0.03 [-0.06; 0.12]
**WC, cm**	0.02 [-0.08; 0.11]	0.01 [-0.09; 0.06]
**Waist-to-hip ratio**	0.00 [-0.00; 0.00]	0.00 [-0.00; 0.00]
**SBP, mmHg**	0.09 [-0.11; 0.30]	0.04 [-0.16; 0.25]
**DBP, mmHg**	0.12 [-0.01; 0.24]	0.08 [-0.05; 0.21]
**Glucose, mmol/l**	0.00 [-0.01; 0.01]	-0.00 [-0.01; 0.00]
**HbA1c, mmol/l**	0.00 [-0.02; 0.01]	0.00 [-0.00; 0.01]
**Cholesterol, mmol/l**	-0.00 [-0.02; 0.01]	-0.00 [-0.02; 0.01]
**HDL-C, mmol/l**	-0.01 [-0.01; -0.001]	-0.01 [-0.01; -0.00]*
**LDL-C, mmol/l**	0.00 [-0.01; 0.01]	-0.00 [-0.01; 0.01]
**Triglycerides, mmol/l**	0.01 [-0.01; 0.02]	0.00 [-0.01; 0.02]
**ARIC**	0.04 [-0.04; 0.12]	0.03 [-0.06; 0.11]
**SCORE**	0.01 [-0.03; 0.05]	0.00 [-0.04; 0.04]

ARIC, Atherosclerosis Risk In Communities; DBP, diastolic blood pressure; HbA1c, glycated haemoglobin; HDL-C, high-density lipoprotein cholesterol; LDL-C, low-density lipoprotein cholesterol; SBP, systolic blood pressure; SCORE, Systematic COronary Risk Evaluation; WC, waist circumference.

Model 1: Unadjusted model.

Model 2: Adjusted for age, gender, education level and total physical activity.

*Indicates significant association, p < 0.05.

After adjustment for age, gender, education level and total physical activity, overall sedentary time was weakly but significantly related to fasting plasma levels of HDL-C.

Table 3 shows the prospective associations of TV time, computer time, reading time and time spent on other sedentary behaviours (such as passive transport and talking with friends) and cardiometabolic risk. TV time was weakly but significantly associated with only one of the 13 cardiometabolic risk markers (i.e. HDL-C) whereas reading time, computer time and time spent on other sedentary behaviours were not associated with any of the studied risk factors.

**Table 3:** **Prospective associations (Beta and 95% CI) between self-reported sedentary time (h/day) in four domains**^**a**^**and cardiometabolic risk factors**^**b**^

	**TV time**	**PC time**	**Reading time**	**Other sedentary time**
	**Beta [95% CI]**	**Beta [95% CI]**	**Beta [95% CI]**	**Beta [95% CI]**
**Weight, kg**	0.11 [-0.10; 0.32]	0.06 [-0.23; 0.35]	0.06 [-0.25; 0.36]	-0.07 [-0.30; 0.16]
**WC, cm**	-0.03 [-0.25; 0.20]	0.04 [-0.24; 0.31]	-0.08 [-0.44; 0.28]	0.00 [-0.23; 0.23]
**Waist-to-hip ratio**	-0.00 [-0.00; 0.00]	0.00 [-0.00; 0.00]	-0.00 [-0.01; 0.00]	0.00 [-0.00; 0.00]
**SBP, mmHg**	-0.22 [-0.64; 0.21]	-0.50 [-1.07; 0.11]	-0.06 [-1.05; 0.92]	0.43 [-0.19; 1.05]
**DBP, mmHg**	-0.09 [-0.33; 0.17]	-0.15 [-0.50; 0.20]	0.14 [-0.53; 0.82]	0.07 [-0.26; 0.40]
**Glucose, mmol/l**	-0.01 [-0.03; 0.00]	-0.00 [-0.02; 0.02]	0.02 [-0.02; 0.05]	-0.01 [-0.03; 0.01]
**HbA1c, mmol/l**	-0.00 [-0.01; 0.01]	0.01 [-0.01; 0.02]	-0.01 [-0.03; 0.01]	-0.00 [-0.02; 0.01]
**Cholesterol, mmol/l**	-0.01 [-0.04; 0.01]	-0.01 [-0.05; 0.03]	-0.03 [-0.07; 0.02]	0.01 [-0.02; 0.05]
**HDL-C, mmol/l**	-0.01 [-0.02; -0.00]*	0.00 [-0.01; 0.01]	0.00 [-0.01; 0.02]	-0.00 [-0.01; 0.01]
**LDL-C, mmol/l**	-0.01 [-0.03; 0.01]	-0.01 [-0.04; 0.03]	-0.04 [-0.08; 0.02]	0.02 [-0.02; 0.06]
**Triglycerides, mmol/l**	0.02 [-0.00; 0.04]	0.00 [-0.03; 0.03]	0.04 [-0.01; 0.09]	-0.01 [-0.04; 0.01]
**ARIC**	-0.04 [-0.20; 0.13]	-0.01 [-0.26; 0.27]	-0.13 [-0.27; 0.02]	-0.05 [0.19; 0.29]
**SCORE**	-0.03 [-0.12; 0.07]	-0.11 [-0.26; 0.04]	-0.14 [-0.48; 0.20]	0.15 [-0.00; 0.30]

ARIC, Atherosclerosis Risk In Communities; DBP, diastolic blood pressure; HbA1c, glycated haemoglobin; HDL-C, high-density lipoprotein cholesterol; LDL-C, low-density lipoprotein cholesterol; SBP, systolic blood pressure; SCORE, Systematic COronary Risk Evaluation; WC, waist circumference.

^**a**^TV viewing, computer use, reading and other sedentary behaviours.

^b^All associations were adjusted for age, gender, education and total physical activity. In addition, the models were adjusted for time spent sedentary in the other three domains.

*Indicates significant association, p < 0.05.

## Discussion

This study examined the prospective association of leisure time spent on various sedentary activities with cardiometabolic risk factors in adults. Our findings demonstrate that there are almost no prospective associations between overall sedentary time or sedentary time in different domains (i.e. TV viewing, computer use, reading and other sedentary behaviours) with individual or clustered cardiometabolic risk factors.

Our finding that higher levels of overall sedentary time were negatively associated with HDL-C over a two-year period was weak as the regression coefficient for this association was small. This is only partly in line with previous studies examining objectively measured sedentary time and cardiometabolic risk among individuals at increased risk for T2DM. Henson et al. [24] found a significant association between objectively assessed sedentary time and HDL-C as well as 2 hr glucose, but they found no association with waist circumference, BMI, fasting glucose and HbA1c among middle aged and older adults at high risk of impaired glucose regulation. Ekelund et al. [25] found no associations between objectively assessed sedentary time and fasting plasma insulin among individuals with a parental history of T2DM.

Separate analyses for TV time revealed a statistically significant association with only one out of 13 cardiometabolic risk factors studied (i.e. HDL-C). The lack of consistent associations with cardiometabolic risk is in line with a previous study of our group [10], but in contrast to findings of others [11],[13],[14]. Pinto Pereira et al. demonstrated a significant association between TV time and increase in BMI [14] and six out of nine cardiometabolic risk factors in women and four out of nine in men [13]. Heinonen et al. [11] found significant associations of TV time with waist circumference and BMI. Among adults at increased T2DM risk, Ekelund et al. [25] found significant associations of TV time with fasting insulin and insulin resistance (HOMA-IR) at baseline, but not follow-up.

We found no associations of computer time with any of the cardiometabolic risk factors. This finding is in line with Altenburg et al. [10], but in contrast to Heinonen et al. [11], who found significant associations with waist circumference and BMI in women but not men.

In line with Heinonen et al. [11], we found no significant association between reading time and indicators of overweight. To date, no studies have been published on the separate association of reading time with cardiometabolic risk factors, T2DM and CVD and risk scores. The limited time spent on reading in this population might be an explanation for the lack of an association.

Time spent on sedentary behaviours other than TV viewing, computer use and reading was not related to cardiometabolic risk factors. Unfortunately, except for the examples given (e.g. sitting while talking with friends, playing board games, sitting in the car), we have no specific information on the sedentary behaviours in this category. Therefore, sedentary time in this domain, as well as total sedentary time, might be underestimated. Future studies should further examine less frequently explored sedentary behaviours including passive transport, sitting during meals and relaxing, in order to obtain a complete overview of sedentary time. Additionally, sedentary behaviours using electronic devices such as phones and tablets should be further explored.

One important difference with the previous studies mentioned above is that we adjusted for total physical activity (i.e. light, moderate and vigorous) instead of moderate-to-vigorous physical activity only. This is important when examining the true association between sedentary time and cardiometabolic risk [26]. Our finding that there is virtually no association between sedentary time and cardiometabolic risk is in line with Maher et al. [26], who demonstrated that potential (weak) associations disappear when analyses are adjusted for total physical activity. Another explanation for the lack of an association of self-reported sedentary time with cardiometabolic risk may be the limited variance in sedentary time over the 2-year follow-up period. Furthermore, similar to physical activity that is generally overestimated [27], sedentary time may be underestimated especially when assessed in one question. Adding up sedentary time in different domains, i.e. in separate questions, may result in a more accurate estimate of actual sedentary time, when covering all sedentary behaviours.

The prospective design with four measurements over 2 years is an important strength of the present study. The distinction between different domains of sedentary behaviour (i.e. TV viewing, computer use, reading and other sedentary behaviours) further strengthens our study. Although self-reported measures of sedentary behaviour are sensitive to recall bias, these measures are required to study the association of different types of sedentary behaviours and cardiometabolic risk factors. The AQuAA questionnaire correlated moderately on test-retest reliability assessment (ICC = 0.60, CI = [0.40; 0.74]) regarding time spent on sedentary behaviours [21]. However, correlation between the AQuAA and Actigraph was low and non-significant (Spearman correlation coefficient = 0.15). Finally, the category of time spent in sedentary behaviours other than TV viewing, computer use and reading is vague and may not cover all other sedentary behaviours.

We conclude that sedentary time (overall and in different domains) was virtually not prospectively associated with cardiometabolic risk among adults at increased cardiometabolic risk. Future studies, using more accurate measures of different sedentary behaviours, should further examine the possible distinct association of different sedentary behaviours and cardiometabolic risk factors.

## Abbreviations

ARIC: Atherosclerosis Risk In Communities

AQuAA: Activity Questionnaire for Adolescents and Adults

CVD: Cardiovascular disease

GEE: Generalized Estimating Equations

HbA1c: Insulin glycated haemoglobin

HDL-C: High-density lipoprotein cholesterol

LDL-C: Low-density lipoprotein cholesterol

LPL: Lipoprotein lipase

SCORE: Systematic COronary Risk Evaluation

SQUASH: Short QUestionnaire to ASsess Health-enhancing physical activity

T2DM: Type 2 diabetes mellitus

## Competing interests

The authors declare that they have no competing interests.

## Authors’ contributions

TA was involved in the conception and the design of the study, analysis, data interpretation, drafting and manuscript writing. JL was involved in the conception and design of the study, data acquisition, data interpretation and critically revising the manuscript. SB, GN and MC were involved in data interpretation and critically revising the manuscript. All authors read and approved the final manuscript.
